# Crystal structure and absolute configuration of (4*S*,5*R*,6*S*)-4,5,6-trihy­droxy-3-methyl­cyclo­hex-2-enone (gabosine H)

**DOI:** 10.1107/S2056989017004509

**Published:** 2017-03-28

**Authors:** Gaurao D. Tibhe, Mario A. Macías, Enrique Pandolfi, Valeria Schapiro, Leopoldo Suescun

**Affiliations:** a Departamento de Química Orgánica, Facultad de Química, Universidad de la República, Montevideo 11800, Uruguay; b Department of Chemistry, Universidad de los Andes, Cra 1 N° 18A-12, 111711 Bogotá, Colombia; c Cryssmat-Lab./DETEMA, Facultad de Química - Universidad de la República, Av. Gral. Flores 2124, Montevideo 11800, Uruguay

**Keywords:** crystal structure, absolute configuration, Mitsunobu inversion reaction, natural product

## Abstract

The mol­ecule of the title keto carbasugar, C_7_H_10_O_4_, is formed by a cyclo­hexene skeleton with an envelope conformation, substituted by carbonyl, methyl and hydroxyl groups. The crystal structure is controlled mainly by a combination of strong O—H⋯O and weak C—H⋯O hydrogen bonds, forming nearly perpendicular chains running parallel to the [110] and [-110] directions. This perpendicularity is caused by a tetra­gonal pseudosymmetry influenced by the similarity between the *a* and *b* axes, the value of 90.9770 (10)° of the β angle and the action of a 2_1_ screw axis, which transform each chain into its corresponding nearly orthogonal one.

## Abstract 2

The absolute configuration of the title compound, determined as 4*S*,5*R*,6*S* on the basis of the synthetic pathway, was confirmed by single-crystal X-ray diffraction. The mol­ecule is formed by a substituted six-membered cyclo­hexene ring adopting an envelope conformation and substituted by carbonyl, methyl and hydroxyl groups. The supra­molecular structure is mainly built by a combination of O—H⋯O and weaker C—H⋯O hydrogen bonds.

## Chemical context   

Gabosines are regarded as secondary metabolites and were first isolated in 1974 from *Streptomyces strains* (Tsushiya *et al.*, 1974 ▸)*.* These compounds are closely related to carbasugars and exhibit DNA binding properties (Tang *et al.*, 2000 ▸). To date, 15 gabosines have been isolated, of which 14 have been synthesized. Gabosine H is one of such kind, whose total synthesis has recently been achieved by our research group (Tibhe *et al.*, 2017 ▸), starting from a biotransformation of toluene that introduces chirality. A further sequence of reactions, including Mitsunobu and final removal of the acetyl protective group, led to the title compound.
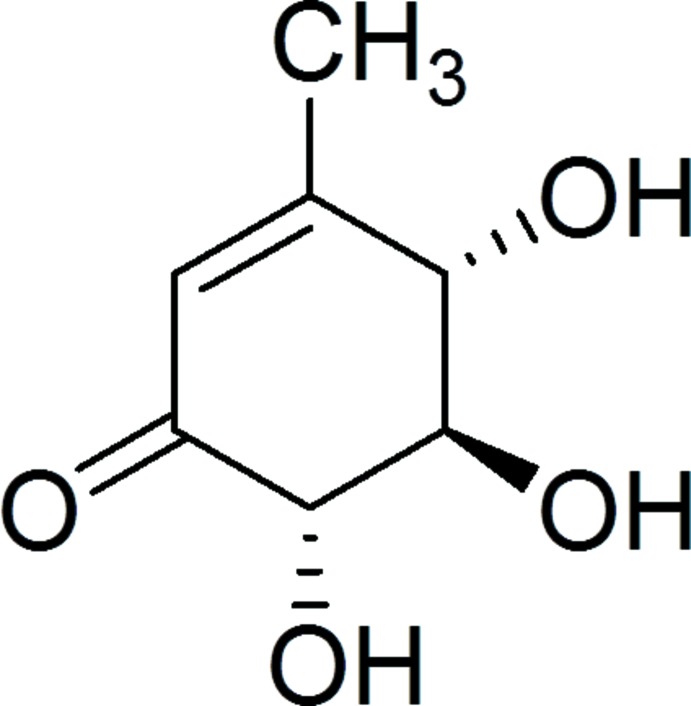


## Structural commentary   

**Figure 1:**
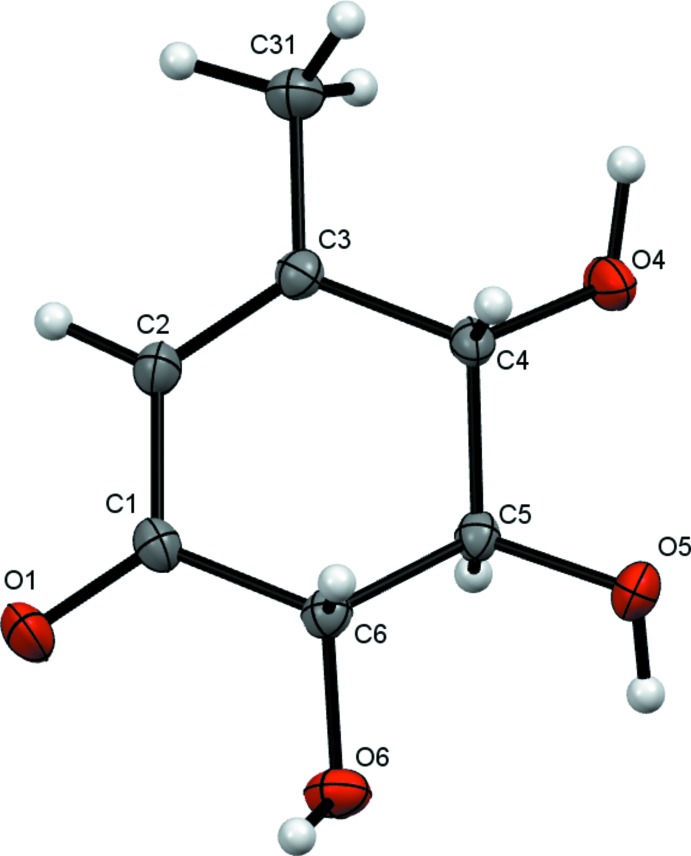
The mol­ecular structure of the title compound, showing the anisotropic displacement ellipsoids drawn at the 50% probability level.

Fig. 1 ▸ shows the mol­ecule of the title compound. The absolute configuration of gabosine H with the carbonyl, methyl and hydroxyl groups in equatorial positions, determined as 4*S*,5*R*,6*S* on the basis of synthetic pathway, was confirmed by X-ray diffraction on the basis of anomalous dispersion of light atoms only. The six-membered ring (C1–C6) in the mol­ecule adopts an envelope conformation with atom C5 as the flap [deviating from the plane through the other ring atoms by 0.639 (2) Å] and puckering parameters *Q* = 0.4653 (19) Å, *θ* = 129.5 (2)° and *φ* = 66.7 (3)°.

## Supra­molecular features   

**Figure 2:**
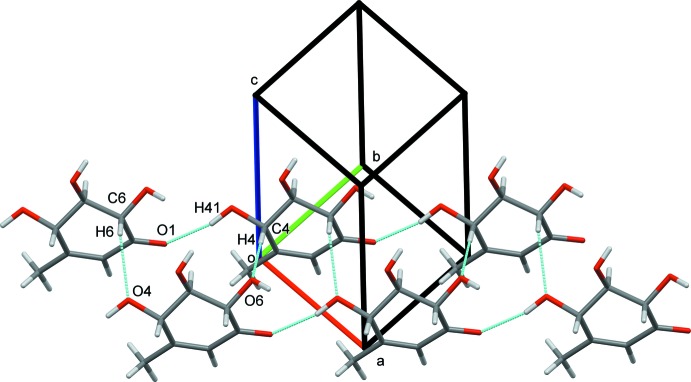
Partial crystal packing of the title compound showing the C—H⋯O and O—H⋯O hydrogen bonds (dotted lines) along [110] and [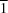
10], forming sheets parallel to the (001) plane.

**Figure 3:**
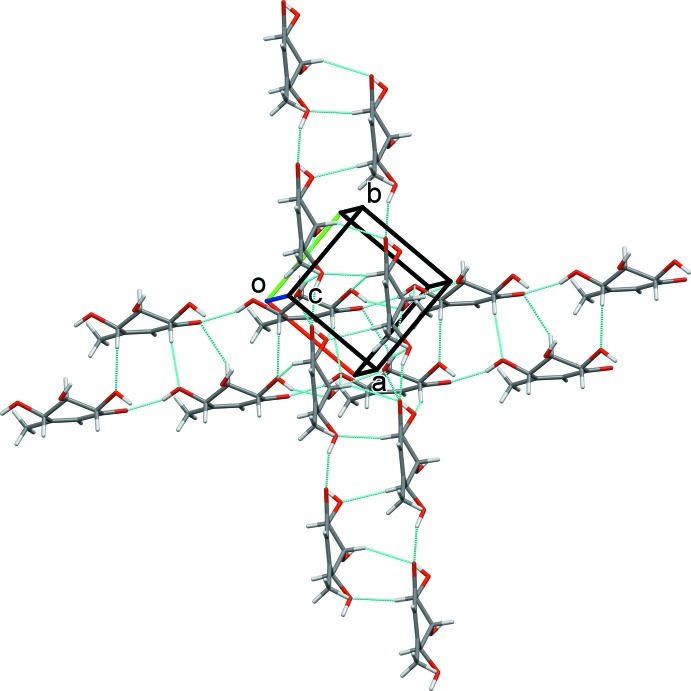
Partial crystal packing of the title compound connected into a nearly orthogonal assembly along [001] through C—H⋯O and O—H⋯O hydrogen bonds (dotted lines).

In the crystal structure, hydrogen bonds O4—H41⋯O1^i^ [symmetry code: (i) *x* − 1, *y* − 1, *z*] link the mol­ecules into chains that run along the [110] direction (Table 1 ▸). These chains are further connected by weaker C6—H6⋯O4^ii^ and C4—H4⋯O6^iii^ [symmetry codes: (ii) *x* + 1, *y*, *z*; (iii) *x*, *y* − 1, *z*] hydrogen bonds along the [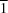
10] direction, forming (001) sheets (Fig. 2 ▸). Considering that the chains run along the diagonal of the *ab* plane and the fact that *a*≃*b*, it is possible to observe that the *2*_1_ screw axis parallel to *b* transforms each chain into a nearly orthogonal one along [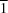
10] (Fig. 3 ▸). The orthogonal chains are connected by single C6—H6⋯O6^iv^, O6—H61⋯O5^v^ and bifurcated O5—H51⋯O4^vi^ and O5—H51⋯O5^vi^ hydrogen bonds [symmetry codes: (iv) −*x* + 1, *y* − 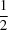, −*z* + 1; (v) −*x* + 1, *y* + 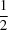; (vi) −*x*, *y* + 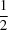, −*z* + 1] to define a three-dimensional array along the [001] direction. These hydrogen bonds connect the orthogonal chains by pairs along [001]. Between these neighboring [001] sheets, weak dipolar or van der Waals forces stabilize the assembly along the *c-*axis direction.

## Database survey   

A search of the Cambridge Structural Database (CSD Version 5.36 with one update; Groom *et al.*, 2016 ▸) was carried out considering mol­ecular structures similar to gabosine and its derivatives. Among the natural compounds, only the structure of gabosine N, (4*R*,5*R*,6*R*)-4,5,6-trihy­droxy-2-methylcyclo­hex-2-enone (Tang *et al.* 2000 ▸), has been reported. The remaining hits were mainly derivatives of other gabosines different from H or derivatives such as 5-hy­droxy-4-methyl-7-oxabi­cyclo­[4.1.0]hept-3-en-2-one (White *et al.*, 2010 ▸), which is an epoxide with different configuration of the asymmetric carbons compared with gabosine H.

## Synthesis and crystallization   

**Figure 4:**
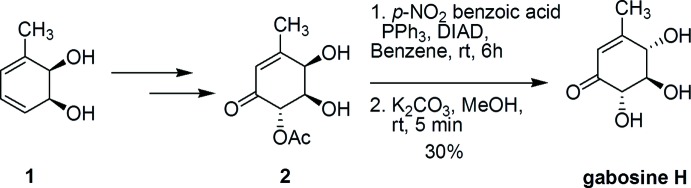
Reaction scheme.

The synthesis of gabosine H was achieved by inversion of the allylic –OH group using Mitsunobu conditions followed by deprotection. (4*R*,5*R*,6*S*)-5-Acet­oxy-4,5-dihy­droxy-3-methyl­cyclo­hex-2-enone (**2**, Fig. 4 ▸; 0.149 mmol, 0.030 g) was dissolved in 1 ml of benzene and TPP (0.283 mmol, 0.078 g) was added along with *p*-nitro­benzoic acid (0.299 mmol, 0.050 g) and diisopropyl azodi­carboxyl­ate (DIAD; 0.297 mmol, 0.060 g). The reaction mixture was stirred at room temperature for 6 h. The solvent was evaporated and the crude mass was used for the next reaction without further purification. The crude product was dissolved in MeOH (3.4 mL), a catalytic qu­antity of K_2_CO_3_ was added and the reaction mixture was stirred at room temperature for 5 min and filtered. Evaporation of the solvent from the filtrate afforded crude gabosine H, which was purified by column chromatography (CH_2_Cl_2_/MeOH, 9:1 *v*/*v*) to afford pure gabosine H as a white crystalline powder (yield: 7.2 mg, 30%; m.p. 390.6 K. Suitable crystals for X-ray analysis were obtained by dissolving the solid in a minimum amount of methanol and allowing it to evaporate at room temperature. IR (KBr): 3400, 2875, 1660 cm^−1. 1^H NMR (400 MHz, CD_3_OD): δ = 2.07 (*s*, 3 H), 3.56 (*dd*, *J* = 10.8, 2.4 Hz, 1 H), 4.01 (*d*, *J* = 10.8 Hz, 1 H), 4.23 (*d*, *J* = 8.4 Hz, 1 H), 5.92 (*s*, 1 H).

## Refinement   

Crystal data, data collection and structure refinement details are summarized in Table 2 ▸. H atoms bonded to C were placed in calculated positions (C—H = 0.93–0.98 Å) and included as riding contributions with isotropic displacement parameters set to 1.2–1.5 times the *U*_eq_ of the parent atom. Hy­droxy H atoms were located in difference density maps and were refined with *U*_iso_(H) = 1.5 *U*_eq_(O). The absolute structure parameter *y* was calculated using *PLATON* (Spek, 2009 ▸). The resulting value of 0.07 (7) indicates that the absolute structure was determined correctly (Hooft *et al.*, 2008 ▸).

## Supplementary Material

Crystal structure: contains datablock(s) I. DOI: 10.1107/S2056989017004509/rz5209sup1.cif

Structure factors: contains datablock(s) I. DOI: 10.1107/S2056989017004509/rz5209Isup2.hkl

Supporting information file. DOI: 10.1107/S2056989017004509/rz5209Isup3.cml

CCDC reference: 1539327

Additional supporting information:  crystallographic information; 3D view; checkCIF report

## Figures and Tables

**Table 1:** Hydrogen-bond geometry (Å, °)

*D*—H⋯*A*	*D*—H	H⋯*A*	*D*⋯*A*	*D*—H⋯*A*
O4—H41⋯O1^i^	0.94 (4)	1.96 (4)	2.873 (2)	163 (3)
C6—H6⋯O4^ii^	0.98	2.46	3.195 (2)	131
C4—H4⋯O6^iii^	0.98	2.36	3.345 (3)	179
C6—H6⋯O6^iv^	0.98	2.62	3.306 (2)	127
O6—H61⋯O5^v^	0.87 (4)	1.99 (4)	2.811 (2)	155 (3)
O5—H51⋯O4^vi^	0.85 (4)	2.45 (3)	3.050 (2)	128 (3)
O5—H51⋯O5^vi^	0.85 (4)	2.26 (4)	3.0402 (12)	152 (3)

Symmetry codes: (i) 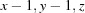; (ii) 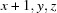; (iii) 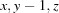; (iv) 
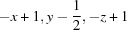
; (v) 
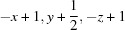
; (vi) 
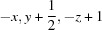
.

**Table 2:** Experimental details

Crystal data	
Chemical formula	C_7_H_10_O_4_
*M*_r_	158.15
Crystal system, space group	Monoclinic, *P*2_1_
Temperature (K)	298
*a*, *b*, *c* (Å)	5.4143 (2), 5.4176 (2), 11.9200 (5)
β (°)	90.977 (1)
*V* (Å^3^)	349.59 (2)
*Z*	2
Radiation type	Cu *K*α
μ (mm^−1^)	1.06
Crystal size (mm)	0.37 × 0.34 × 0.10

Data collection	
Diffractometer	Bruker D8 Venture/Photon 100 CMOS
Absorption correction	Multi-scan (*SADABS*; Bruker, 2013 ▸)
*T*_min_, *T*_max_	0.588, 0.754
No. of measured, independent and observed [*I* > 2σ(*I*)] reflections	12187, 1492, 1472
*R*_int_	0.046
(sin θ/λ)_max_ (Å^−1^)	0.637

Refinement	
*R*[*F* ^2^ > 2σ(*F* ^2^)], *wR*(*F* ^2^), *S*	0.033, 0.088, 1.08
No. of reflections	1492
No. of parameters	111
No. of restraints	1
H-atom treatment	H atoms treated by a mixture of independent and constrained refinement
Δρ_max_, Δρ_min_ (e Å^−3^)	0.25, −0.18
Absolute structure	Flack *x* determined using 639 quotients [(*I* ^+^)−(*I* ^−^)]/[(*I* ^+^)+(*I* ^−^)] (Parsons *et al.*, 2013 ▸)
Absolute structure parameter	0.09 (11)

Computer programs: *SAINT* (Bruker, 2013 ▸), *SHELXS2014* (Sheldrick, 2008 ▸), *SHELXL2014* (Sheldrick, 2015 ▸), *Mercury* (Macrae *et al.*, 2008 ▸).

## supplementary crystallographic information

### Crystal data

**Table:**

C_7_H_10_O_4_	*F*(000) = 168
*M**_r_* = 158.15	*D*_x_ = 1.502 Mg m^−^^3^
Monoclinic, *P*2_1_	Cu *K*α radiation, λ = 1.54178 Å
*a* = 5.4143 (2) Å	Cell parameters from 9784 reflections
*b* = 5.4176 (2) Å	θ = 3.7–78.8°
*c* = 11.9200 (5) Å	µ = 1.06 mm^−^^1^
β = 90.977 (1)°	*T* = 298 K
*V* = 349.59 (2) Å^3^	Parallelepiped, colorless
*Z* = 2	0.37 × 0.34 × 0.10 mm

### Data collection

**Table:**

Bruker D8 Venture/Photon 100 CMOS diffractometer	1492 independent reflections
Radiation source: Cu Incoatec microsource	1472 reflections with *I* > 2σ(*I*)
Detector resolution: 10.4167 pixels mm^-1^	*R*_int_ = 0.046
\j and ω scans	θ_max_ = 79.1°, θ_min_ = 3.7°
Absorption correction: multi-scan (SADABS; Bruker, 2013)	*h* = −6→6
*T*_min_ = 0.588, *T*_max_ = 0.754	*k* = −6→6
12187 measured reflections	*l* = −15→14

### Refinement

**Table:**

Refinement on *F*^2^	Hydrogen site location: mixed
Least-squares matrix: full	H atoms treated by a mixture of independent and constrained refinement
*R*[*F*^2^ > 2σ(*F*^2^)] = 0.033	*w* = 1/[σ^2^(*F*_o_^2^) + (0.0546*P*)^2^ + 0.0518*P*] where *P* = (*F*_o_^2^ + 2*F*_c_^2^)/3
*wR*(*F*^2^) = 0.088	(Δ/σ)_max_ < 0.001
*S* = 1.08	Δρ_max_ = 0.25 e Å^−^^3^
1492 reflections	Δρ_min_ = −0.18 e Å^−^^3^
111 parameters	Extinction correction: SHELXL2014 (Sheldrick, 2015), Fc^*^=kFc[1+0.001xFc^2^λ^3^/sin(2θ)]^-1/4^
1 restraint	Extinction coefficient: 0.161 (15)
Primary atom site location: structure-invariant direct methods	Absolute structure: Flack *x* determined using 639 quotients [(*I*^+^)-(*I*^-^)]/[(*I*^+^)+(*I*^-^)] (Parsons *et al.*, 2013)
Secondary atom site location: difference Fourier map	Absolute structure parameter: 0.09 (11)

### Special details

**Table:**

Geometry. All esds (except the esd in the dihedral angle between two l.s. planes) are estimated using the full covariance matrix. The cell esds are taken into account individually in the estimation of esds in distances, angles and torsion angles; correlations between esds in cell parameters are only used when they are defined by crystal symmetry. An approximate (isotropic) treatment of cell esds is used for estimating esds involving l.s. planes.

### Fractional atomic coordinates and isotropic or equivalent isotropic displacement parameters (Å^2^)

**Table:**

	*x*	*y*	*z*	*U*_iso_*/*U*_eq_	
C1	0.4992 (3)	0.2954 (4)	0.22586 (16)	0.0293 (4)	
C2	0.3815 (4)	0.1293 (4)	0.14457 (16)	0.0313 (5)	
H2	0.4304	0.1373	0.0702	0.038*	
C3	0.2068 (3)	−0.0338 (3)	0.17173 (15)	0.0262 (4)	
C31	0.0921 (4)	−0.2029 (5)	0.08715 (17)	0.0374 (5)	
H31A	0.1711	−0.1813	0.0163	0.056*	
H31B	−0.0807	−0.1656	0.0790	0.056*	
H31C	0.1119	−0.3706	0.1116	0.056*	
C4	0.1234 (3)	−0.0631 (3)	0.29155 (15)	0.0249 (4)	
H4	0.2190	−0.1965	0.3269	0.030*	
C5	0.1658 (3)	0.1719 (4)	0.35829 (15)	0.0237 (4)	
H5	0.0534	0.3005	0.3302	0.028*	
C6	0.4314 (3)	0.2607 (3)	0.34819 (14)	0.0258 (4)	
H6	0.5418	0.1368	0.3818	0.031*	
O1	0.6472 (3)	0.4524 (3)	0.19769 (14)	0.0453 (5)	
O4	−0.1316 (3)	−0.1192 (3)	0.29994 (14)	0.0398 (4)	
H41	−0.172 (6)	−0.273 (8)	0.269 (3)	0.060*	
O5	0.1120 (3)	0.1206 (3)	0.47319 (11)	0.0336 (4)	
H51	0.096 (5)	0.263 (7)	0.502 (3)	0.050*	
O6	0.4549 (3)	0.4821 (3)	0.40965 (13)	0.0374 (4)	
H61	0.606 (7)	0.485 (7)	0.437 (3)	0.056*	

### Atomic displacement parameters (Å^2^)

**Table:**

	*U*^11^	*U*^22^	*U*^33^	*U*^12^	*U*^13^	*U*^23^
C1	0.0252 (8)	0.0305 (10)	0.0322 (9)	−0.0046 (8)	0.0038 (7)	0.0021 (8)
C2	0.0322 (9)	0.0360 (11)	0.0257 (8)	−0.0051 (8)	0.0055 (7)	−0.0002 (8)
C3	0.0251 (8)	0.0263 (9)	0.0274 (8)	0.0013 (7)	0.0018 (6)	−0.0011 (7)
C31	0.0429 (11)	0.0358 (11)	0.0335 (10)	−0.0084 (10)	0.0007 (8)	−0.0050 (9)
C4	0.0241 (8)	0.0219 (9)	0.0289 (9)	0.0001 (7)	0.0061 (6)	0.0011 (7)
C5	0.0227 (8)	0.0242 (9)	0.0243 (8)	0.0024 (6)	0.0037 (6)	0.0007 (6)
C6	0.0240 (8)	0.0267 (10)	0.0267 (8)	0.0011 (7)	0.0004 (6)	−0.0019 (7)
O1	0.0464 (9)	0.0459 (10)	0.0440 (8)	−0.0236 (8)	0.0080 (7)	−0.0002 (7)
O4	0.0287 (8)	0.0363 (9)	0.0548 (9)	−0.0099 (6)	0.0152 (6)	−0.0124 (7)
O5	0.0363 (7)	0.0388 (8)	0.0260 (7)	−0.0005 (6)	0.0091 (5)	−0.0016 (6)
O6	0.0329 (7)	0.0364 (9)	0.0429 (8)	−0.0016 (6)	−0.0044 (6)	−0.0125 (7)

### Geometric parameters (Å, º)

**Table:**

C1—O1	1.220 (3)	C4—C5	1.517 (2)
C1—C2	1.461 (3)	C4—H4	0.9800
C1—C6	1.521 (2)	C5—O5	1.432 (2)
C2—C3	1.338 (3)	C5—C6	1.523 (2)
C2—H2	0.9300	C5—H5	0.9800
C3—C31	1.490 (3)	C6—O6	1.410 (2)
C3—C4	1.514 (2)	C6—H6	0.9800
C31—H31A	0.9600	O4—H41	0.94 (4)
C31—H31B	0.9600	O5—H51	0.85 (4)
C31—H31C	0.9600	O6—H61	0.87 (4)
C4—O4	1.419 (2)		
			
O1—C1—C2	121.89 (18)	O4—C4—H4	108.6
O1—C1—C6	121.36 (18)	C3—C4—H4	108.6
C2—C1—C6	116.75 (16)	C5—C4—H4	108.6
C3—C2—C1	123.26 (17)	O5—C5—C4	107.89 (15)
C3—C2—H2	118.4	O5—C5—C6	110.15 (14)
C1—C2—H2	118.4	C4—C5—C6	110.99 (14)
C2—C3—C31	122.04 (17)	O5—C5—H5	109.3
C2—C3—C4	121.40 (16)	C4—C5—H5	109.3
C31—C3—C4	116.52 (16)	C6—C5—H5	109.3
C3—C31—H31A	109.5	O6—C6—C1	111.83 (16)
C3—C31—H31B	109.5	O6—C6—C5	107.72 (14)
H31A—C31—H31B	109.5	C1—C6—C5	110.99 (14)
C3—C31—H31C	109.5	O6—C6—H6	108.7
H31A—C31—H31C	109.5	C1—C6—H6	108.7
H31B—C31—H31C	109.5	C5—C6—H6	108.7
O4—C4—C3	113.27 (15)	C4—O4—H41	113 (2)
O4—C4—C5	106.33 (14)	C5—O5—H51	103 (2)
C3—C4—C5	111.22 (14)	C6—O6—H61	106 (2)
			
O1—C1—C2—C3	−175.5 (2)	O4—C4—C5—C6	−175.86 (15)
C6—C1—C2—C3	5.4 (3)	C3—C4—C5—C6	−52.12 (19)
C1—C2—C3—C31	−179.29 (19)	O1—C1—C6—O6	28.4 (3)
C1—C2—C3—C4	−1.9 (3)	C2—C1—C6—O6	−152.56 (18)
C2—C3—C4—O4	145.37 (18)	O1—C1—C6—C5	148.7 (2)
C31—C3—C4—O4	−37.2 (2)	C2—C1—C6—C5	−32.2 (2)
C2—C3—C4—C5	25.7 (2)	O5—C5—C6—O6	−62.1 (2)
C31—C3—C4—C5	−156.85 (17)	C4—C5—C6—O6	178.49 (14)
O4—C4—C5—O5	63.37 (19)	O5—C5—C6—C1	175.17 (16)
C3—C4—C5—O5	−172.89 (14)	C4—C5—C6—C1	55.8 (2)

### Hydrogen-bond geometry (Å, º)

**Table:**

*D*—H···*A*	*D*—H	H···*A*	*D*···*A*	*D*—H···*A*
O4—H41···O1^i^	0.94 (4)	1.96 (4)	2.873 (2)	163 (3)
C6—H6···O4^ii^	0.98	2.46	3.195 (2)	131
C4—H4···O6^iii^	0.98	2.36	3.345 (3)	179
C6—H6···O6^iv^	0.98	2.62	3.306 (2)	127
O6—H61···O5^v^	0.87 (4)	1.99 (4)	2.811 (2)	155 (3)
O5—H51···O4^vi^	0.85 (4)	2.45 (3)	3.050 (2)	128 (3)
O5—H51···O5^vi^	0.85 (4)	2.26 (4)	3.0402 (12)	152 (3)

Symmetry codes: (i) *x*−1, *y*−1, *z*; (ii) *x*+1, *y*, *z*; (iii) *x*, *y*−1, *z*; (iv) −*x*+1, *y*−1/2, −*z*+1; (v) −*x*+1, *y*+1/2, −*z*+1; (vi) −*x*, *y*+1/2, −*z*+1.
